# Use of Muscle Graft for Hemostasis in a Traumatic Transverse Sinus Injury Following a Motorcycle Accident: A Neurosurgical Case Report

**DOI:** 10.7759/cureus.88783

**Published:** 2025-07-25

**Authors:** Karl Francis Y Chan, Guillermo Victorino T Liabres, John Albert Dy

**Affiliations:** 1 Neurosurgery, The Medical City, Pasig, PHL; 2 Neurosurgery, Quirino Memorial Medical Center, Quezon City, PHL; 3 Neurosurgery, Makati Medical Center, Makati, PHL

**Keywords:** acute subdural hematoma, coagulation in neurosurgery, hemostasis, thrombin release, venous sinus injury

## Abstract

Injury to the dural venous sinuses poses a risk for significant hemorrhage and air embolism during surgery. The objectives for this case report are to describe the challenges of a traumatic transverse sinus injury following a motorcycle accident, demonstrate the use of a macerated autologous muscle graft for achieving hemostasis in a venous sinus defect, and highlight key considerations in the perioperative management and postoperative outcomes of patients with cerebral venous sinus injuries. The report describes the surgical intervention and use of macerated muscle tissue for hemostasis of a traumatic transverse sinus injury in a 39-year-old male due to a motorcycle accident. The injury to the sinus is due to an avulsed cortical vein, causing a round defect on the sinus wall. It discusses the technique to repair the sinus and its mechanism. The patient’s postoperative course, recovery, and the rationale behind the chosen surgical approach are discussed as well.

## Introduction

Traumatic injuries to the cerebral venous sinuses leading to a sizable hematoma are rare, with an incidence rate of 0.3%. These injuries, however, are clinically significant due to the potential for massive hemorrhage and exsanguination [1] and the risk of air embolism, which can be life-threatening [2]. These sinus injuries are commonly due to high-energy trauma. Prompt neurosurgical evacuation is essential in patients showing signs of neurological decline caused by mass effect. Additionally, timely control of hemorrhage is important. Venous sinus injury can lead to massive blood loss in a short amount of time. This case shows the surgical considerations and technique used in the management of such an injury.

## Case presentation

A 39-year-old male, the sole rider of a motorcycle wearing a helmet, not intoxicated, was involved in a side-to-side collision with a sport utility vehicle (SUV). The patient did not lose consciousness immediately post-collision and was able to go home. Twelve hours later, he developed a headache, and after 16 hours, his sensorium deteriorated, necessitating emergency room (ER) admission.

Course in the emergency room

Upon arrival at the ER, the patient was initially assessed and found to be hypertensive with a blood pressure of 170/90 mmHg and tachycardic with a heart rate of 100-110 beats per minute. The patient had good chest rise, and his oxygen saturation was at 97%. A cervical collar was applied as a precautionary measure to immobilize his neck. The patient's Glasgow Coma Scale (GCS) score was 6 (E1V1M4). His pupils were 3 mm bilaterally, equal in diameter, and briskly reactive to light. He had intact doll’s eye reflexes and brisk corneal reflexes. The patient had a periorbital hematoma on the left. There were no other overt signs of facial injuries or cerebrospinal fluid (CSF) leak. He was subsequently intubated for airway protection.

**Figure 1:**
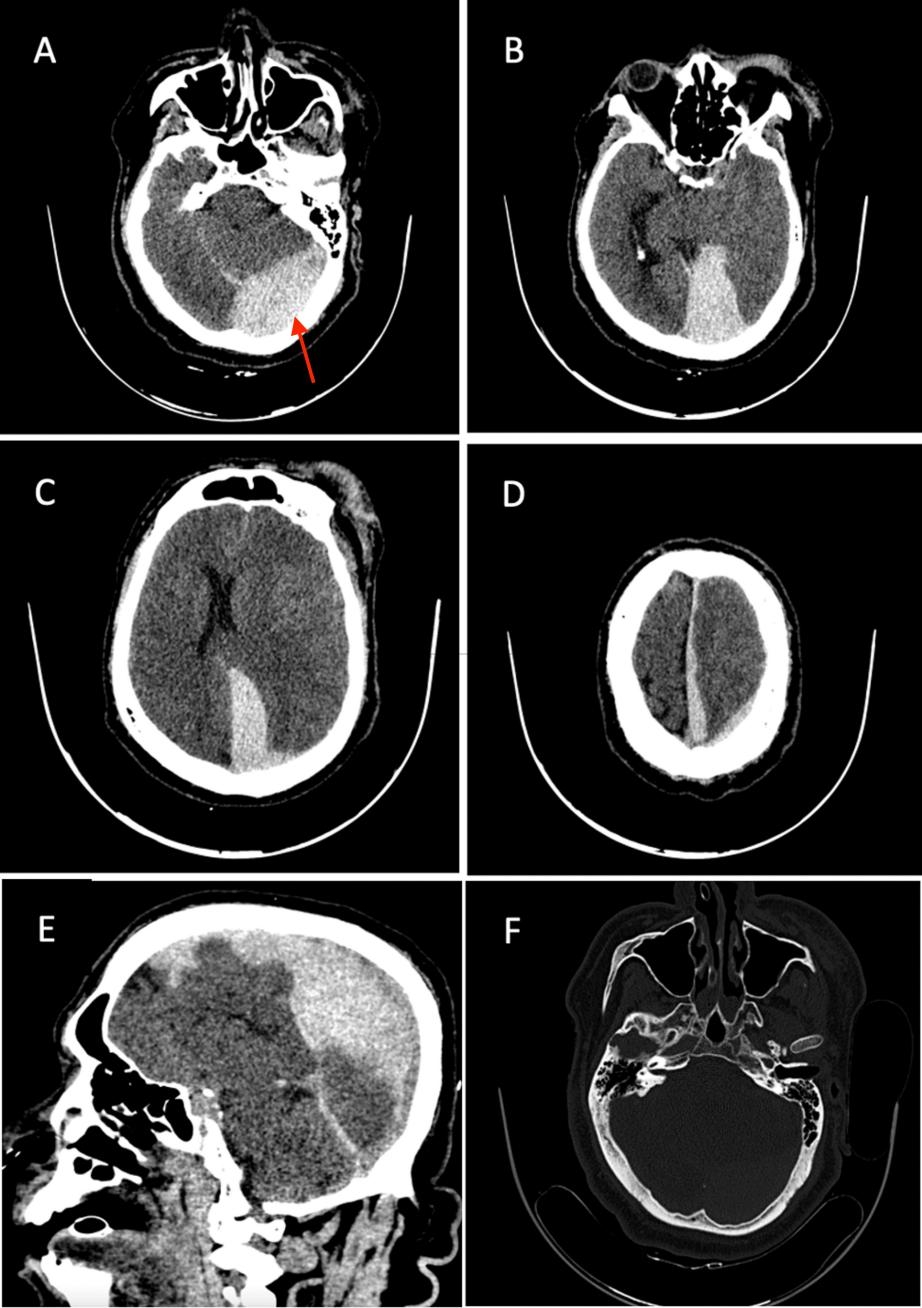
Preoperative plain head CT scan A-D) Axial view showing the acute subdural hematoma in the tentorial and interhemispheric region. Cerebral edema present on the left frontotemporoparietal area with midline shift of 14mm. E) Sagittal view. F) Bone window, level of torcula with no skull fracture. The red arrow indicates the location of the hematoma near the left transverse sinus, suggestive of venous sinus injury, which was confirmed intraoperatively.

The patient was hemodynamically stable and thus was promptly brought to the radiology department for a plain cranial CT scan (shown in Figure 1), which revealed an acute subdural hematoma (ASDH) located on the left occipitoparietal convexity, as well as the occipitoparietal interhemispheric and tentorial areas. The hematoma's maximal thickness was 18 mm within the interhemispheric region, 6 mm over the convexity, and 13 mm at the tentorial area. Additionally, hypodensity observed in the left frontoparietotemporal cortico-subcortical regions was indicative of edema. Midline shift was significant at 14 mm. Effacement of the perimesencephalic cisterns was noted, along with the absence of the left atrium and the occipital and temporal horns. The third ventricle appeared effaced, and there was dilation of the right temporal horn. There were no fractures on the calvarium or skull base. Findings necessitated immediate neurosurgical intervention.

Surgical intervention

The patient underwent a left decompressive hemicraniectomy, evacuation of the hematoma, and repair of a left transverse sinus injury. The indications are based on the patient’s preoperative imaging and clinical presentation. The intraoperative course unfolded as follows.

Incision and Craniotomy

**Figure 2:**
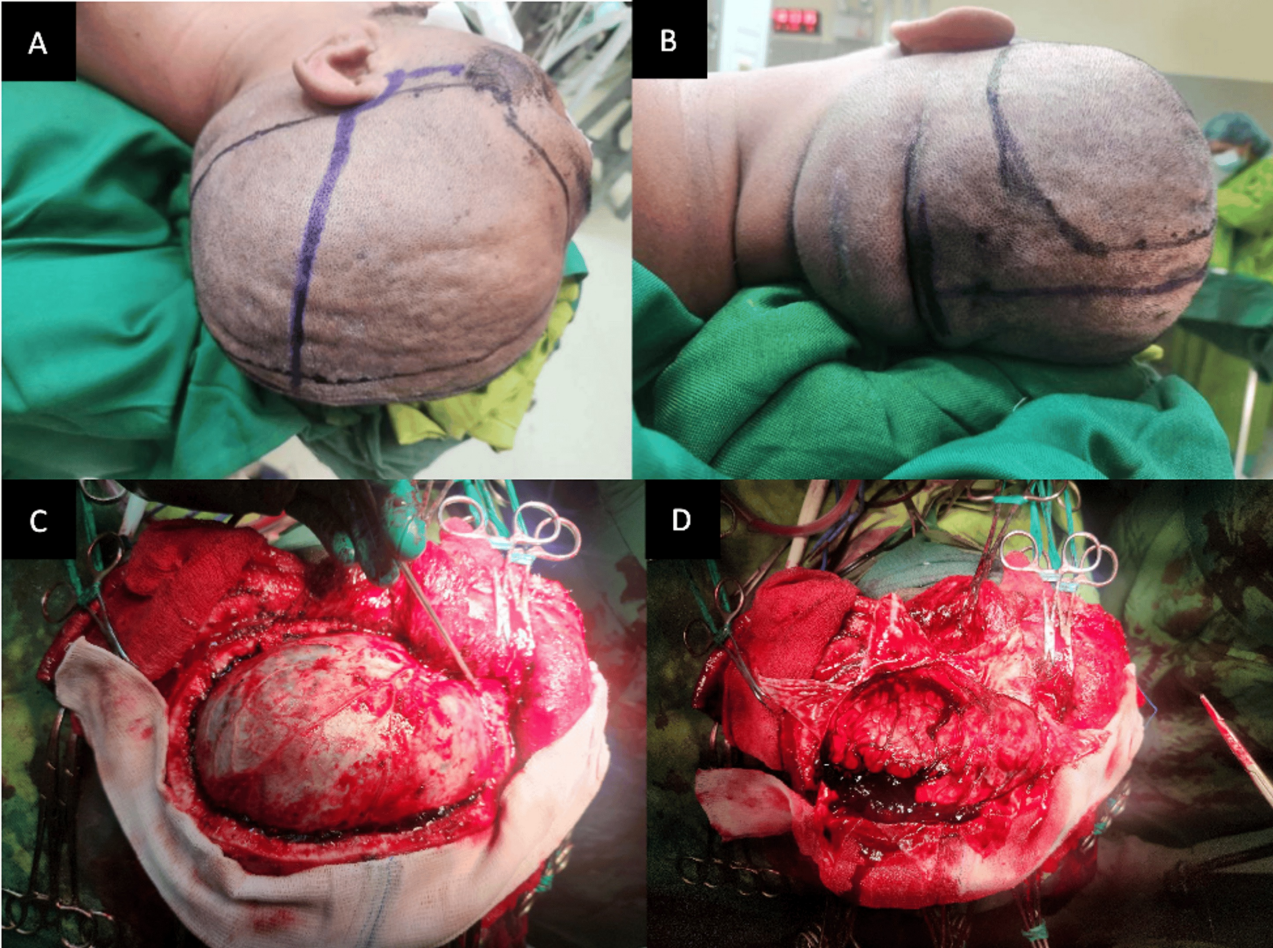
Incision and craniotomy A-B) A T-shaped incision, one limb from the midline behind the hairline to the inion and the other limb from the root of the zygoma to the coronal suture (Kempe’s incision). C) A large frontotemporoparietal craniectomy was done, and tense dura was noted. D) Stellate-fashion durotomy.

A Kempe incision (shown in Figure 2) was utilized to reach the occipital lobe while preserving blood supply to the scalp. A decompressive hemicraniectomy was performed on the left side, ensuring an adequate subtemporal craniectomy. After the removal of the bone flap, the dura was observed to be tense. There was no epidural hematoma observed. The dura was opened in a cruciate fashion, revealing an ASDH over the left occipitoparietal convexity. The brain was noted to bulge out of the craniectomy approximately 5 mm past the outer table.

Evacuation of Hematoma

The ASDH located at the occipitoparietal convexity was carefully evacuated using suction and irrigation. This was followed by the removal of hematoma in the interhemispheric fissure and then the tentorial area. The evacuation process involved meticulous suction and irrigation to avoid damage to the surrounding brain.

Identification and Repair of Transverse Sinus Injury

During the procedure, there was an approximately 2 mm defect on the meningeal layer of the left transverse sinus midway between the torcula and the transverse-sigmoid junction. It was round and actively bleeding. Initial attempts to control the bleeding involved applying a 2 × 2 cm patch of absorbable hemostatic agent and a cottonoid patty for tamponade. While maintaining the tamponade, muscle with fascia was harvested from the temporalis, anticipating its use for a more definitive repair.

Muscle Graft Repair

The cottonoid patty, when removed after several minutes, revealed ongoing bleeding from the sinus injury. The harvested muscle graft was then macerated and placed over the defect, with the edges sutured to the adjacent dura using silk 4-0 interrupted sutures to secure the graft in place. This method promotes hemostasis through the release of thrombin from the macerated muscle. After applying additional absorbable hemostatic patches, gelatin sponge, and a cottonoid patty over the repair site, hemostasis was eventually achieved after removal of the cottonoid patty. The surgical field was inspected for any remaining signs of bleeding. There was no cortical vein identified to be another source of bleeding.

Estimated Blood Loss (EBL) and Operation Time

The EBL was 1000 mL. The operation lasted 3.5 hours. No intraoperative hypotension was noted. One unit of packed red blood cells was transfused.

Postoperative course

Improvements were noted immediately post-surgery, with the patient's GCS improving to 11 (E4VtM6) on the first postoperative day. He was extubated by day 4 and discharged on day 8, with a follow-up indicating full neurological recovery and a GCS of 15 two weeks post-operation. Cranioplasty was discussed with the patient to be performed electively at a later time.

The patient followed up three weeks postoperatively at the outpatient clinic. He was GCS 15, conversant with no neurologic deficits.

## Discussion

The decision to perform a decompressive hemicraniectomy and evacuate the hematoma was made owing to the depressed sensorium as well as the significant mass effect seen on imaging with the aim to alleviate intracranial pressure [3]. The postoperative recovery of the patient validates the effectiveness of the intervention in managing such injuries.

Traumatic venous sinus injuries are noted for their rarity and the surgical challenges they present. Despite advancements in neurosurgical techniques, the principles of venous sinus repair have remained consistent, emphasizing the importance of achieving hemostasis and preserving venous outflow. The use of autologous muscle grafts in sinus repair is supported by their role in promoting thrombin release and subsequent clot formation, a crucial aspect of achieving durable repair. Skeletal muscle myosin promotes the generation of thrombin [4]. Moreover, muscle patches have been successfully used in vascular and sinus repair in neurosurgery, especially when initial attempts using synthetic agents or hemostatic agents alone did not prove to be effective [2].

In a retrospective study by Elkhalek et al., bleeding in compound depressed skull fractures over dural venous sinuses was managed with Gelfoam compression (55%) and direct dural suturing with Gelfoam reinforcement (25%) [5]. Only 5% of their cases used a free muscle graft, which was part of dural closure and not for repairing the sinus wall directly [5]. In contrast, the present case, involving a full-thickness defect in the transverse sinus, used a macerated autologous muscle graft - sutured to the adjacent dura - for patching the defect and for hemostasis. This approach combined the physical tamponade of the graft with the hemostatic effects of muscle myosin, which promotes thrombin formation [4].

In the same study, two patients developed postoperative venous sinus thrombosis (5%), both of whom had their injuries repaired using pericranium patches [5]. One later developed benign intracranial hypertension. In our case, there were no signs of thrombosis or neurologic decline during the recovery period. While this may reflect anatomical differences or patient-specific factors, the use of muscle may have played a role in maintaining sinus patency. The muscle graft likely helped seal the defect while minimizing turbulence that could promote clot formation within the sinus. More importantly, it was readily available and easy to prepare intraoperatively, requiring no additional materials or harvest from distant sites.

In comparison, Rish reported 10 cases involving sinus injuries repaired with autologous venous grafts or pericranial patches [6]. These were applied to the sagittal and transverse sinuses. They used vascular surgical techniques including shunting, irrigation with heparinized saline, and vein grafts. While successful in many cases, these procedures were time-consuming and required microsurgical expertise. The approach used in our case is simpler and potentially more feasible in emergency situations, especially in centers where advanced microsurgical tools or vascular instruments may not be readily available.

Kapp et al. also described an internal shunt technique used to maintain blood flow and reduce venous pressure while repairing a sinus injury [7]. Their method involved inserting a modified pediatric endotracheal tube into the sinus, inflating its balloon tips to temporarily control bleeding. This allowed them to complete a saphenous vein patch repair with less blood loss and better visualization. While innovative, the technique required equipment not routinely available in most operating rooms. In our case, bleeding was controlled with simple tools - cottonoid tamponade, muscle graft, and suturing - without the need for shunting or specialized vascular access. This further supports the practicality of the method used in this case, especially for trauma teams dealing with high-flow sinus injuries in urgent settings.

Ultimately, each of these cases highlights the wide range of strategies available for managing sinus injuries. From simple compression to complex vein grafting and shunting, techniques must be tailored to the anatomy, resources, and urgency of each situation. In this case, the use of a macerated muscle graft was effective in achieving hemostasis, avoided the need for synthetic materials, and minimized the risk of thrombosis. The technique is not new, but its application here supports its value as a reliable and accessible option when synthetic agents or advanced vascular tools are not available or effective.

One risk associated with venous sinus injuries is air embolism during the surgery [8]. When the venous sinus is compromised, there is an increased risk of air entering the venous system. To lessen this risk, preventive measures are taken during surgery. These include lowering the head below the level of the heart to decrease negative pressure, flooding the site with saline, and ensuring meticulous closure of the sinus defect. Furthermore, the use of techniques to stabilize the injured sinus and prevent exposure to air can help reduce this risk.

The management of venous sinus injuries includes performing a venogram. In this case, it was not performed preoperatively. In hindsight, the location of the hematoma on the plain scan should raise suspicion that bleeding may come from a venous sinus. A venogram performed after surgery is an essential step to check for any stenosis or thrombosis of the injured sinus [9]. This imaging will provide critical insights into the success of the repair and help identify any complications that might require further intervention [2].

The defect on the meningeal layer of the transverse sinus, causing bleeding into the subdural space, is likely due to an avulsed draining vein into the sinus. Intraoperatively, there was no such vein identified to be bleeding; however, the shape of the defect was round, hinting at a possible avulsion of a cortical bridging vein. In particular, the inferior cerebral veins drain into the basal sinuses or the deep venous system. They are small-caliber veins that, when sacrificed, have little to no consequence if they do not contribute significantly to the Labbe system [10].

This case emphasizes the application of the established technique in managing hemorrhage from a traumatic transverse sinus injury. The use of muscle tissue for the repair of the sinus injury is significant for its biological characteristics in promoting hemostasis. The muscle graft not only provides a physical barrier over the sinus defect but also actively contributes to the formation of a stable clot, reducing further bleeding and the risk of rebleeding.

## Conclusions

This case discusses the techniques done to repair the transverse sinus injury using a macerated muscle graft from the temporalis muscle. The graft’s effectiveness is attributed to its ability to release thrombin, thereby promoting clot formation and achieving hemostasis. The repair of the transverse sinus defect minimized further potential blood loss intraoperatively. This report emphasizes the importance of anticipating possible venous sinus involvement in hematomas that present in atypical locations. The surgical technique outlined here offers a practical approach that may be valuable in resource-limited settings. Furthermore, the case highlights the versatility and reliability of autologous tissue for controlling bleeding in neurosurgery. Muscle grafts, as demonstrated in this case, provide an alternative when conventional hemostatic agents are unavailable or insufficient. The patient’s favorable outcome and rapid recovery support the feasibility of this technique in acute surgical settings. Ultimately, this case adds to the literature in managing venous sinus injuries and reinforces the role of autologous tissue as a safe and effective option in such scenarios.
